# Food-Derived Nanoscopic Drug Delivery Systems for Treatment of Rheumatoid Arthritis

**DOI:** 10.3390/molecules25153506

**Published:** 2020-07-31

**Authors:** Dong Han, Qilei Chen, Hubiao Chen

**Affiliations:** School of Chinese Medicine, Hong Kong Baptist University, Hong Kong 999077, China; 19424078@life.hkbu.edu.hk

**Keywords:** food nanotechnology, dietary therapeutics, drug delivery, rheumatoid arthritis

## Abstract

Rheumatoid arthritis (RA) is a severe systemic inflammatory disease with no cure at present. Recent developments in the understanding of inflammation and nanomaterial science have led to increased applications of nanostructured drug delivery systems in the treatment of RA. The present review summarizes novel fabrications of nanoscale drug carriers using food components as either the delivered drugs or carrier structures, in order to achieve safe, effective and convenient drug administration. Polyphenols and flavonoids are among the most frequently carried anti-RA therapeutics in the nanosystems. Fatty substances, polysaccharides, and peptides/proteins can function as structuring agents of the nanocarriers. Frequently used nanostructures include nanoemulsions, nanocapsules, liposomes, and various nanoparticles. Using these nanostructures has improved drug solubility, absorption, biodistribution, stability, targeted accumulation, and release. Joint vectorization, i.e., using a combination of bioactive molecules, can bring elevated therapeutic outcomes. Utilization of anti-arthritic chemicals that can self-assemble into nanostructures is a promising research orientation in this field.

## 1. Introduction

Rheumatoid arthritis (RA) is a common, serious and chronic autoimmune inflammatory disease. It is characterized by chronic multi-joint synovitis, which often leads to damage to articular cartilage, bone, tendons, and ligaments and subsequent loss of joint function [1]. The global prevalence of RA is approximately 1%, with the prevalence in women approximately three times that of men. RA can occur at any age; however, prevalence is particularly high in people between 40 and 60 years of age [2]. The disease is associated with several co-morbidities, such as atherosclerosis-associated cardiovascular disease, infection, malignancy, pulmonary disease, osteoporosis, and depression. These comorbidities lead to a high mortality rate in RA patients [3].

RA is a complex disease whose etiology remains unclear. Its pathogeneses are complex and multifactorial, involving genetic, environmental, biological, and biomechanical factors. Such factors might together alter the threshold for immune activation or failed regulation, promoting joint inflammation [1]. At this time, there is no cure for RA. In order to manage disease development, there are four main types of drug treatments, each with clinical limitations. Firstly, glucocorticoids (GCs), e.g., prednisolone (PRD) and dexamethasone (DEX), are strong immunosuppressive agents yet are prone to result in serious side effects including osteonecrosis and atherosclerosis [4]. Secondly, non-steroidal anti-inflammatory drugs (NSAIDs), e.g., celecoxib and indomethacin, function by inhibiting cyclooxygenase-2 (COX-2) and tend to cause gastrointestinal diseases as well as cardiovascular toxicities [5]. Thirdly, disease-modifying anti-rheumatic drugs (DMARDs), including methotrexate (MTX), are immunosuppressive but are slow to act. Therefore they are often prescribed in combination with other drugs; however, they may cause ulcers, hepatotoxicity, pneumonia, and other adverse effects. Fourthly, biological drugs, such as cytokine antagonists, have high efficacy in the early stages of the disease but suffer from short half-lives, high costs, and tendencies to induce severe bacterial infections, pneumonia, and tuberculosis [6].

Nanoscopic drug delivery systems derived from food components are a new approach for safe, effective, and convenient treatment of RA. Some food products have anti-arthritic activities but are limited in clinical application because of low absorption via the oral route; some are suitable to be used as structuring agents of safe and stable drug carriers due to special physicochemical properties. The nano-size enables drug distribution in systemic circulation without capillary entrapment and removal by the mononuclear phagocyte system (MPS). Furthermore, these structures facilitate adsorption along the gastrointestinal (GI) tract due to their high surface-to-volume ratio, improving drug bioavailability [7]. Amphiphilic drug carriers can enhance solubility of hydrophobic drugs by incorporating the drug molecule into an oil phase. Core-shell nanostructures protect the drug molecule from physiological and enzymatic degradation, contributing to drug stability [8]. Delivery systems with specific surface modification in specific sites (e.g., the inflamed joints) result in targeted drug delivery [9].

The present review focuses on recent applications of nanoscopic drug delivery systems utilizing food components. Various nanocarriers are discussed, especially lipid-based ones. Food components, including functional small compounds and macromolecules, have been adopted in such nanostructures as delivered drugs or as carrier materials. Perspectives on food-derived nanoscopic drug delivery in clinical management of RA are also discussed.

## 2. Food Components as Delivered Drugs to Treat RA

### 2.1. Polyphenols

#### 2.1.1. Curcumin

**Figure 1:**
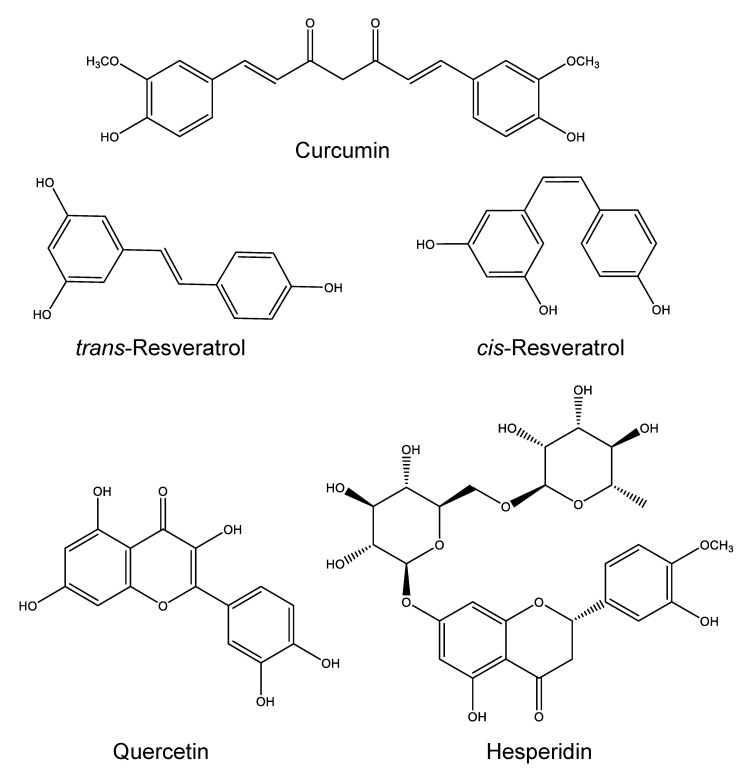
Representative food components as anti-RA (rheumatoid arthritis) agents in nanoscopic drug delivery systems.

Curcumin is a natural polyphenol isolated from the rhizome of turmeric (*Curcuma longa*), a signature ingredient in curry, and a representative food-derived chemical with anti-RA efficacy (Figure 1). It acts against RA by inhibiting various pro-inflammatory signaling molecules, including those in the MAPK/RANK/c-Fos/NFATc1 pathways [10]. The utility of curcumin as a therapeutic agent is seriously limited by its poor aqueous solubility (0.0004 mg/mL), extensive hepatic metabolism, low gastrointestinal absorption (less than 25%), and rapid systemic elimination [11]. A major strategy for curcumin bioavailability enhancement is to improve the compound’s solubility and stability by encapsulating it in a lipid core, covered with either hydrophobic or hydrophilic walls. Under this approach, curcumin has been applied in various nano-formulations and administered orally, topically, and parenterally, in all cases demonstrating enhanced anti-arthritic effects.

**Figure 2:**
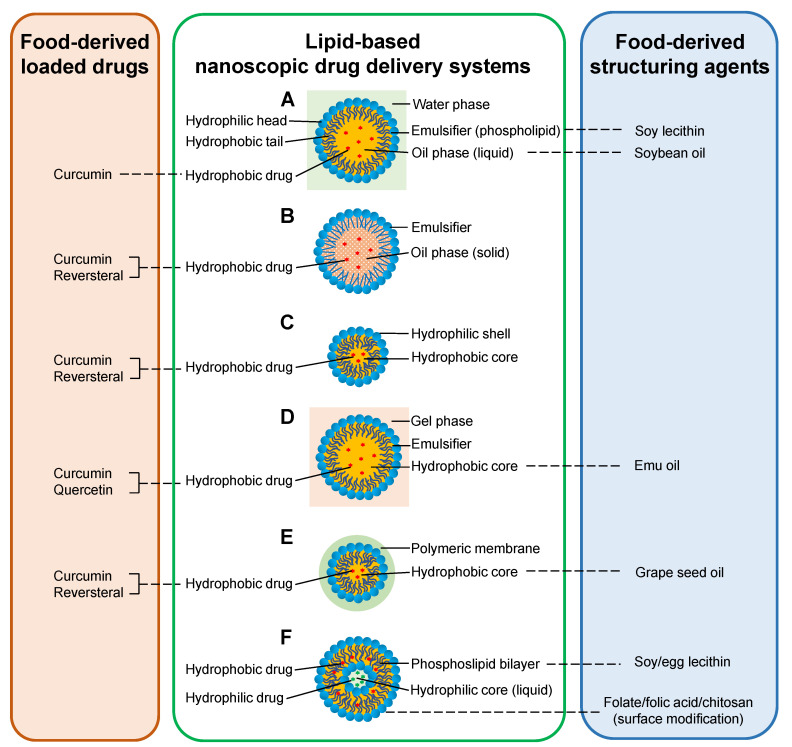
Representative lipid-based nanoscopic drug delivery systems with food-derived chemicals as loaded drugs and/or structuring agnets. (**A**) Nanoemulsion; (**B**) solid lipid nanoparticle; (**C**) nanomicelle; (**D**) nanoemulsion gel; (**E**) nanocapsule; (**F**) liposome.

Nano-formulations that can improve oral bioavailability of curcumin include nanoemulsions (NEs), solid lipid nanoparticles (SLNs), and nanomicelles. NEs are generally heterogeneous systems consisting of an oil phase, a water phase, and an emulsifier, with drug substances dissolved in liquid oil droplets (typically less than 100 nm) and then dispersed in the water phase (Figure 2A). The expansion of an emulsifier is necessary for producing droplets with smaller size because it reduces the interfacial tension and surface energy in unit range between the oil and water phases of the emulsion. NEs show advantages in high drug-loading capacity [12], core-shell protection of embedded drugs from physicochemical and enzymatic degradation [8], wide drug distribution along the GI tract, prolonged drug release due to large surface area [7], and ease of preparation [13]. A threefold increase in maximum plasma concentration and the area under the plasma drug concentration-time curve (AUC) was observed when comparing curcumin in NEs to curcumin suspensions. Oral administration of curcumin-NEs significantly decreased TNF-α and IL-1β levels in both synovial fluid and blood serum (Table 1) [13]. It is therefore concluded that the formulation of NEs significantly enhanced curcumin absorption.

SLNs are colloidal carriers derived from oil-in-water (O/W) emulsions by replacing the oil phase with solid lipids (Figure 2B) [14]; they have been proposed to effectively increase absorption and reduce clearance of the encapsulated drugs [15]. When SLNs are delivered orally, they can carry drugs into the lymph, avoiding removal by the liver [16]. The solid lipid matrix is able to protect the encapsulated compounds against exposure to enzymatic degradation [17]. Surfactants used in preparation of SLNs, e.g., Tween 80 and lecithin, contribute towards increased permeability of the intestinal membrane to the loaded chemicals [18]. The nano-sized particles (120–200 nm) not only have increased surface area, facilitating high bio-adhesion to the GI wall and subsequently prolonged uptake, but also bypass MPS pickup, avoiding in vivo metabolism and elimination of the embedded drugs [19]. A study confirmed such improved delivery provided by SLNs: rats with complete Freund’s adjuvant-induced arthritis (AIA) orally treated with curcumin-loaded SLNs exhibited enhanced antioxidant, anti-inflammatory, and immunomodulatory effects on the joint synovia [20].

Micelles are self-aggregated by amphiphilic monomers; the assembled supramolecular globular structures have hydrophobic cores that can trap drugs with poor water solubility, and hydrophilic shells that can protect the encapsulated drugs from gastrointestinal enzymes and physiological pH [21]. Nanomicelles have particle sizes within 10–200 nm (Figure 2C); they are sufficiently large to prevent premature elimination via globular filtration, and are small enough to permeate blood vessels [22]. The nanostructure can also improve cellular uptake of the drugs by facilitating internalization via endosomes [23]. Therefore, nanomicelles enable prolonged circulation, reduced administered dose, and diminished toxicity of therapeutics. Such advantages of nanomicelles have been proved by a clinical trial in which RA patients orally undertaking the curcumin nanomicelles exhibited positive changes in joint scores, tender joint count, and swollen joint count [24].

Topical delivery of curcumin can be achieved by loading the compound in nanoemulsion gels (NEGs). NEs are beneficial for topical delivery specifically due to their powerful permeation ability and little irritating effect. However, low viscosity restrains their transdermal application. Adding NEs into viscous gel bases to form NEGs can overcome this drawback (Figure 2D) [25]. It was observed that NEG promoted stratum corneum lipid fluidization and subsequent incorporation into the lipid bilayer, enabling deposition of curcumin in the epidermis and dermis; and that the O/W nature of NEG retained the lipophilicity of curcumin to combat the slow progression of the arthritic conditions [26]. Topically delivered curcumin NEG was proved to have evident anti-inflammatory effects on rats with carrageenan-induced paw edema; these effects were comparable to the effects of the reference drug diclofenac and significantly more potent than gel formulation of crystalline curcumin [27]. Curcumin NEG was also observed with ameliorating efficacy on AIA rats [28].

**Figure 3:**
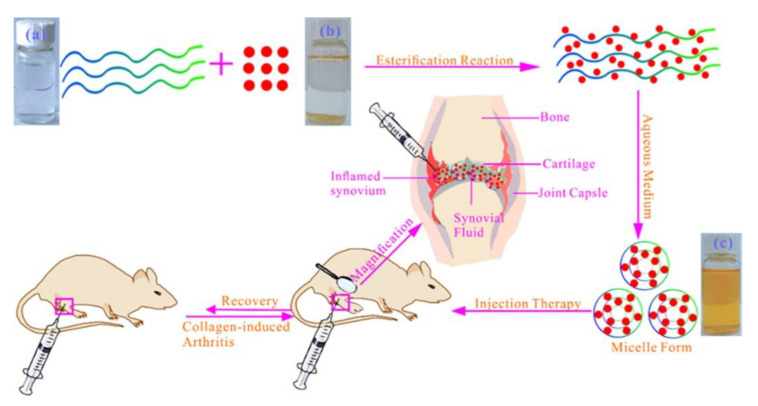
Schematic preparation and administration of drug-loaded nanomicelles. Photos of (**a**) hyaluronic acid, (**b**) curcumin, and (**c**) hyaluronic acid/curcumin [32]. Reproduced with permission from Fan et al., *ACS Appl. Mater. Interfaces*; published by the American Chemical Society, 2018.

Injections of curcumin have been reported with core-shell structures of nanocapsules and nanomicelles. Nanocapsules are vesicular systems in which a drug is confined in an inner core, consisting of lipid or polymer matrix, surrounded by a polymeric membrane (Figure 2E) [29]. There is some evidence of nanocapsulation increasing therapeutic efficacy of the encapsulated drug, as a result of improved drug chemical stability due to isolation from physiological medium, reduced tissue irritation due to biocompatible polymeric shell, and controlled drug release and enhanced drug biodistribution due to the carrier properties [30]. It is reported that AIA rats intraperitoneally injected with curcumin-encapsulated lipid core nanocapsules (LNC) had significantly decreased paw edema with no hepatotoxicity; co-encapsulation of curcumin and reversteral was observed to have even more pronounced anti-edema effects; drugs loaded in LNC showed greater efficacies than drugs in solutions [31]. Another study discovered that rats with intra-articular injection of nanomicelles composed of curcumin and hyaluronic acid, a main extracellular matrix component, were observed to have less edema, decreased joint friction, and diminished biomarker expression compared to rats injected with drug solutions (Figure 3) [32].

#### 2.1.2. Resveratrol

Resveratrol, a natural compound abundant in grapes, peanuts and giant knotweed, has been reported to inhibit arthritis and reduce bone erosion and cartilage damage in animal models (Figure 1) [33]. It has been discovered to act against arthritis by regulating aromatic hydrocarbon receptors, which are known to affect several immune mediators in RA [34,35], and by inhibiting angiogenesis to suppress synovial hyperplasia [36]. Unfortunately, these effects can be hindered by poor solubility and instability of the compound. Amphiphilic poly-lactic acid (PLA) and its derivatives have shown multiple advantages as advanced delivery systems: they can (1) increase solubility of compounds with low polarities by encapsulating them via hydrophobic interactions, (2) avoid side reactions of lipid-based preparations because they are biodegradable and biocompatible when used in situ, and (3) provide sustained drug effects by preventing rapid clearance. Therefore, such polymers are promising materials for use as nano-carriers of resveratrol.

Due to the abovementioned merits of PLA, a PLA-coated co-micellar nanosystem of resveratrol was reported reducing cartilage lesions and synovial inflammation in arthritic rats via intra-articular injection [37]. Moreover, a core-shell nanocomposite was found largely enhancing therapeutic performance of resveratrol. The nanocomposite had a core of quadrilateral ruthenium nanoparticles (QRu NPs) and a shell of dextran sulfate (DS)-modified poly (lactic-co-glycolic acid) (PLGA). With QRu being photothermal, precise drug release can be controlled with an extraneous light source, and photoacoustic imaging can be conducted to provide guidance for the distribution and therapeutic use of the nanomedicine. With PLGA being thermosensitive and amphiphilic, excellent loading capacity of resveratrol can be achieved. DS is a targeting molecule with affinity to the scavenger receptor of macrophages. QRu-PLGA-resveratrol-DS NPs were observed to effectively attenuate RA by accurately inducing M2 macrophage polarization [38].

### 2.2. Flavonoids

#### 2.2.1. Quercetin

Quercetin is widely distributed in frequently consumed foods including apples, onions, cranberries, blueberries, tomatoes, tea and red wine (Figure 1) [39]. The natural flavonoid has been reported to inhibit pro-inflammatory cytokines by suppressing NF-κB signaling [40] and act against angionegesis and synoviocyte proliferation and in arthritic tissues [41], showing high potential as an anti-arthritic agent. Clinical applications of quercetin are nonetheless extremely confined, because the compound has high first-pass metabolism, low skin penetration, rapid excretion, poor water solubility, and low stability [42]. A number of attempts to enhance bioavailability of quercetin using nanoscopic drug delivery systems have been reported, via different administration routes, including oral administration, transdermal application, and joint injection.

When administered orally, quercetin delivery was found to be improved by using the nano-carriers thioglycolic acid-capped cadmium telluride quantum dots (TGA-CdTe QDs). Such QDs encapsulated the compound into QD-quercetin complexes, which exhibited anti-arthritic effects at lower concentrations than free quercetin. The QD–quercetin complexes were observed to have outstanding performance in restoring hematological changes and inducing cartilage regeneration in AIA rats [43].

For transdermal application, skin permeability and physicochemical stability of quercetin was enhanced when administered as a quercetin-loaded NEG. Compared to free quercetin gel, the quercetin-NEG was found with no toxic effect on synoviocytes, stronger inhibitory effect on lipopolysaccharide-induced TNF-α production in macrophages, and more evident inhibition of paw edema in AIA rats [44].

A quercetin delivery system by intra-articular injection was developed by loading the compound in polycaprolactone (PCL) microspheres, which were confirmed biocompatible both in vitro and in vivo. The system not only enabled controlled drug release in joint cavities for more than 31 days, but also drug entrapment efficiency and drug release could be optimized by adjusting PCL concentration [45].

#### 2.2.2. Hesperidin

There is high abundance of hesperidin in peels of orange and lemon. The flavanone glycoside has been demonstrated to inhibit acute and chronic stages of inflammation, and ameliorate clinical statuses of arthritis in various animal models (Figure 1) [46,47]. However, oral administration of hesperidin achieves low therapeutic efficacy due to two major drawbacks: (1) its poor water solubility limits absorption into the body, and (2) its high sensitivity to gastric pH and enzymes makes it unstable in various biological environments.

A study has shown that the absorption, stability, delivery, and therapeutic efficacies of hesperidin can be improved by encapsulating the compound in gum acacia-stabilized green silver NPs (GA-AgNPs) for oral administration [48]. For absorption enhancement, GA, as water-soluble polysaccharides wrapping around the AgNPs, not only increased aqueous solubility of hesperidin but also provided effective drug loading by exposing multiple functional moieties on the AgNPs surfaces. The anionic nature of GA contributed to particle surface negativity, thereby enhancing physical stability, and the nano-range size of the particles prevented drug degradation by plasma proteins and high salt concentrations. Targeted delivery of hesperidin was achieved due to the membrane-crossing capability of AgNPs; drug release was controlled because the compound was surface-tethered. It was discovered that hesperidin-loaded GA-AgNPs exerted evident ameliorating effects on AIA rats via TLR-2 and TLR-4 signaling pathways.

## 3. Food Components as Structuring Agents of Anti-RA Nanocarriers

### 3.1. Soybean Oil

Soybean oil is extracted from the seeds of soybean; it mainly comprises polyunsaturated linoleic acid (51%) and monounsaturated oleic acid (22.6%) [49]. Viscosities of vegetable oils are negatively correlated with amounts of polyunsaturated fatty acids (PUFA) [50]. Due to the relatively high amount of PUFA, soybean oil has a rather low viscosity of 69 mPa·s at 24 °C. In an NE system, an oil phase with lower viscosity enables easier breakup of oil droplets and subsequent formation of smaller particles, enhancing drug biodistribution via circulation [51]. A molecular dynamics simulation further revealed that curcumin as a loaded drug accelerates the self-assembly process of more balanced, symmetrical, and compact soybean oil-based NE systems. Moreover, owing to the oxygen-containing groups of curcumin, polyphenol compounds tend to distribute evenly across the NE surface, thereby gaining the most access to the solvent [52]. Therefore, curcumin has been loaded on soybean-oil-based NEs to enhance its absorption after oral administration (Table 2) [13].

Soybean oil in NE systems can also act as effective drug penetration enhancers for skin delivery, by entering phospholipid bilayers and separating them into different domains. Fatty acids, especially unsaturated ones with *cis* configuration (e.g., linoleic acid, the major component in soybean oil), have a greater perturbing effect on the lipid packing than those with *trans* configuration. It is also speculated that soybean-oil-based NEs can provide higher thermodynamic activity of delivered drugs, and that the NEs preferentially penetrate and accumulate in hair follicles [53]. Due to the permeation-enhancing ability of soybean oil, a hydrogel-thickened NE system, using soybean oil as the oil phase and soy lecithin as one of the surfactants showed significantly increased permeation rates of a lipophilic mixture for topical therapy of arthritis and minor joint and muscle pain [54].

### 3.2. Grape Seed Oil

Grape seed oil contains a high amount of linoleic acid (74.7%), contributing to low viscosity [49]. During nanocapsule formulation, the driving force is rapid diffusion of an oil phase (solubilized with loaded drugs) in an aqueous phase, inducing interfacial nanoprecipitation of a polymer membrane surrounding the oil droplets. Therefore, in general, the lower the viscosity of the oil and the interfacial tension, the smaller the nanocapsules that will be formed, favoring drug delivery [55]. Grape seed oil is also rich in antioxidative polyphenols and tocopherols as well as nutrients including vitamins [56]. As a result, nanocapsules have been created using grape seed oil as the inner core to not only counteract lipid oxidation but also provide the loaded drug with additional health properties. It was observed that grape seed oil based nanocapsules co-encapsulating curcumin and resveratrol have satisfactory storage stability at room temperature for 3 months with unchanged particle size (207–218 nm) and polydispersity index (0.11–0.13) [30], and that the co-encapsulation showed improved in vitro antioxidant effects and anti-edema effects on AIA rats [31].

### 3.3. Emu Oil

Emu oil is extracted from adipose tissue of the emu (*Dromaius novaehollandiae*), a flightless bird indigenous to Australia. It mainly contains oleic acid (52%), linoleic acid (20%), and α-linolenic acid (1–2%) [57]. The oil has outstanding skin penetration efficacy because (1) its high concentration of monosaturated fatty acid (e.g., oleic acid) provides satisfactory fluidity, and (2) its content ratio of linoleic acid and α-linolenic acid is close to that of human skin [58]. An NEG formulation loaded with curcumin was prepared using emu oil and carbopol gel as the oil and gel phases, respectively. The NEG was applied on the dorsal skin of rats, showing evident ameliorating effects on carrageenan-induced paw edema, as well as on AIA rats by alleviating clinical statuses, restoring biochemical changes, and improving joint radiological and histological parameters [28].

Emu oil has also been reported to possess anti-inflammatory properties: it has been extensively used by the Australian aborigines to treat inflamed joints and other inflammatory diseases [57]. Therefore, the oil can act as an adjuvant to treat inflammation-related conditions. Co-administration of emu oil and curcumin via oral route showed significant anti-edema and anti-arthritic effects on acute and chronic rat models, exhibiting a 5.2-fold increase in AUC compared with administration of curcumin alone [59].

### 3.4. Lecithin

#### 3.4.1. Soy Lecithin

Lecithin refers to a mixture of amphiphilic yellow-brown fatty substances from plants and animals, most commonly from soybeans (accounting for over 80% of worldwide production) and egg yolk [60]. It is mainly composed of glycerophospholipids including phosphatidylcholine, phosphatidylethanolamine, phosphatidylinositol, phosphatidylserine, and phosphatidic acid [61]. The phospholipids from lecithin can be used to form liposomes. Liposomes are nano-sized spherical vesicles with at least one lipid bilayer around an aqueous core. This structure makes them compatible with cell membranes, and it means they can incorporate either hydrophilic molecules in the aqueous core or hydrophobic compounds within the lipid bilayer (Figure 2F). Therefore, liposomes have attracted much attention as safe and convenient pharmaceutical carriers [62].

Soy lecithin-based liposome products have been reported to have efficient drug entrapment, controlled drug release, evident drug bioavailability, and high safety. It was calculated that such liposomes can achieve 67.34% encapsulation and 67.26% 24 h release of celecoxib [63]. A parallel study was conducted using topically delivered liposomes loaded with triptolide, a hydrophobic anti-inflammatory diterpenoid epoxide with narrow therapeutic window derived from *Tripterygium wilfordii*, and orally administrated triptolide tablets, on collagen-induced arthritis (CIA) in rats. Compared to the tablet administration, the liposomal treatment exhibited similar therapeutic performances yet significantly lower side effects in the heart, liver, kidney, and stomach [64]. Liposomes made from soy phosphatidylcholine (SPC), a prominent phospholipid found in soy lecithin (19–21% of total weight), were prepared by encapsulating an antioxidant and immunomodulatory 3-phenylcoumarin derivative, showing therapeutic potential to treat neutrophil-mediated inflammatory joint diseases. When applied to immune complex-stimulated neutrophils from healthy human and RA patients, the loaded liposomes not only suppressed the release of neutrophil extracellular traps and chemotaxis in vitro, but also avoided evident toxicity. When applied to rats with zymosan-induced arthritis, the liposomes significantly reduced joint edema and leukocyte infiltration at a low dose (1.5 mg/kg), which was 2.6-fold lower than the dose of DEX as positive control [65].

**Figure 4:**
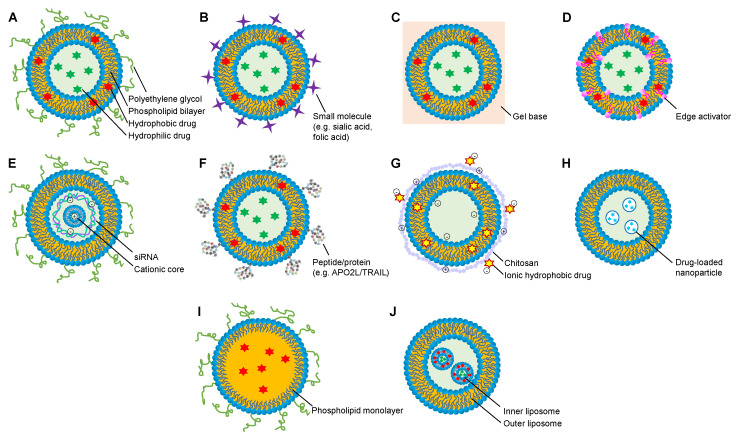
Schematic structures of representative liposome-derived nanoscopic drug delivery systems. (**A**) Sterically stabilized nanoliposome; (**B**) small molecule-modified liposome; (**C**) liposome hydrogel; (**D**) ultradeformable liposome; (**E**) wrapsome; (**F**) peptide/protein-modified liposome; (**G**) chitosan-modified liposome; (**H**) nanoparticle-loaded liposome; (**I**) lipid–polymer hybrid nanoparticle; (**J**) double liposome.

Unsaturated fatty acids such as SPC lack stability against oxidation; hydrogenated SPC (HSPC) can be more stable and thus is more suitable for drug vectorization in complex systems. HSPC has been used to assemble sterically stabilized (pegylated) nanoliposomes (NSSLs) loaded with GCs to achieve improved therapeutic efficacies (Figure 4A). By intravenous injection, the NSSLs demonstrated controlled GC release both systemically (during circulation) and locally (in AIA rat paws), and significantly reduced arthritic severity in AIA rats throughout all disease stages [66]. Both intravenous and subcutaneous administrations of GC-loaded NSSLs significantly suppressed rat arthritis and restored cytokine profiles in rat serum and splenocyte tissue culture; the therapeutic effects were comparable to higher doses of free GCs or with TNF-α antagonists [67]. HSPC-based liposomes with surface modification of sialic acid (Figure 4B) can intrinsically bind to peripheral blood neutrophils (PBNs) during RA progression, because inflamed PBNs have high surface expression of L-selectin [68], which is a selected target of sialic acid [69]. As a result, it was observed that a GC carried by the PBN-targeted liposomes was directed to inflammatory rat joints, exhibiting strong suppression effects on RA [70].

#### 3.4.2. Egg Lecithin

Egg yolk contains a much higher content of lecithin than soybeans (approximately 10% and 2% of total weight, respectively); the major component of egg lecithin is egg phosphatidylcholine (EPC; 80%). In addition, egg lecithin contains higher levels of saturated fatty acids than soy lecithin, resulting in higher oxidative stability [62].

Liposomes made of egg lecithin have been found to have improved properties in terms of slow and controlled release of therapeutic small molecules. It was found that liposomes that mainly consisted of EPC and cholesterol, with inclusion of small amounts of strearylamine and phosphatidyl glycerol, enabled slow release of indomethacin. The slow release may be due to electrostatic interaction and hydrogen bonding between the lipid and the drug. As a result, in rat models, the optimal liposomes showed significantly higher inhibition of edema and one third of ulceration as compared to free drug administration [71]. Moreover, an egg lecithin-based liposome hydrogel patch (LHP) was designed for topical administration of triptolide (Figure 4C). Hind paws of CIA rats were pierced by a microneedle array, and triptolide-loaded LHPs were pasted on the pierced parts. The microneedle-aided local delivery of triptolide eliminated hepatic first-pass metabolism and digestive toxicity of the compound, and provided stable, long-term drug release, showing pharmacodynamics and pharmacokinetic advantages in treating RA [72]. To improve drug permeation during transdermal delivery, ultradeformable liposomes (UDLs) have been developed by incorporating surfactant or “edge activator” into lipid bilayers, resulting in elevated elasticity (Figure 4D) [73]. With the optimum ratio of bilayer matrix (EPC) and an edge activator (Tween 80), a UDL gel entrapping MTX was applied topically on AIA rats, showing 1.5 and 2.15 times higher MTX permeation for 24 h than that of MTX-conventional gel (without Tween 80) and MTX-plain gel, respectively [74].

EPC-made liposomes can also be applied to carry small interfering RNAs (siRNAs) and proteins, as either targeted delivery systems or a therapeutic performance-enhancing strategy to treat RA. Wrapsomes (WSs), a novel form of liposomes, contain siRNA and cationic lipofection complex in a core that is enveloped by a neutral lipid bilayer containing EPC (Figure 4E). With uncharged surface, WSs show several advantages in comparison to common cationic liposomes: (1) WSs tend to escape plasma protein binding and thus can retain pharmacological functions; (2) they resist endothelial cell membrane attachment and MPS entrapment, preventing adverse effects including embolism, a complication reported with the use of cationic liposomes; and (3) surface pegylation of WSs allows long half-life of the complexes in systematic circulation [75]. For instance, WSs loaded with TNF-α siRNA were found to have significantly decreasing arthritic severity in CIA mice via intravenous injection by mainly incorporating into CD11b^+^ macrophages and neutrophils in the inflamed synovium, and silencing TNF-α expression by these cells. Efficient and targeted delivery of siRNAs to arthritic joints by siRNA/WSs means that they have great therapeutic potential [76]. Examples of protein-loaded liposomes include EPC-based large unilamellar vesicles (LUVs; liposomes with single bilayer; 150–200 nm in diameter) with surface binding of APO2L/TRAIL, an apoptosis-inducing ligand from the TNF superfamily (Figure 4F). It was found that infiltrating T lymphocytes in RA synovial fluids are sensitive to APO2L/TRAIL, and that the number of exosomes carrying bioactive APO2L/TRAIL drastically decreases during RA progression [77]. On such basis, the LUVs were designed to supply APO2L/TRAIL in its naturally occurring exosome-associated secreted form to RA synovium, consequently significantly eliminating synovial hyperplasia and reducing inflammatory infiltrate and vascularity without systemic toxicity in vivo. The APO2L/TRAIL-LUVs exhibited higher therapeutic efficacy than soluble APO2L/TRAIL, possibly because the association of APO2L/TRAIL with liposomes increased the protein local concentration, enhancing its receptor crosslinking potential and avoiding protein conformational changes [78].

### 3.5. Chitosan

Chitosan (CHS) is a linear polyaminosaccharide synthesized from deacetylation of chitin, which is the structural element in the exoskeleton of crustaceans, e.g., crabs and shrimp. The amino group in chitosan has a pKa value of approximately 6.5, leading to significant protonation in neutral solution and subsequently increased acidity. This makes chitosan highly water-soluble and ready to bind on negatively charged surfaces such as mucosal membranes [79]. Therefore, this natural polyelectrolyte is widely applied in nanotechnology and drug delivery systems because of its excellent mucoadhesive capability, in addition to its biodegradable, biocompatible, and nontoxic properties.

CHS nanostructures loaded with anti-arthritic agents, e.g., NSAIDs [80], DMARDs [81], GCs [82], natural products [83], and zinc salts [84], have been reported with more potent efficacies than preparations without CHS (Table 3). In order to improve stability and flexibility of CHS NPs, other materials have been incorporated in the preparations. A CHS–lipid hybrid delivery system, CHS-coated nanoliposomes, showed high encapsulation efficiency of indomethacin (99%) and gastro retentive behavior, due to the electrostatic interactions between the cationic CHS and the negatively charged indomethacin and liposomal surface, respectively, holding great potential for stable oral drug delivery (Figure 4G) [80]. Polysialic acid (PSA)-trimethyl CHS (tmCHS) NPs brought out increased in vivo efficacy of entrapped DMARDs, where the carboxyl groups of PSA electrostatically interacted with the ammonium groups of tmCHS, forming nano-sized, stable, and flexible complexes [85].

Some crosslinkers have been utilized to improve release properties of the CHS-based nanoformulations. Malic acid was used to covalently crosslink the CHS chain, forming stable colloid systems, which can retard drug release by preventing medium penetration and slowing down drug diffusion into the medium [82]. Stearic acid (SA), which has a long acyl chain and negative surface charge, can form amphiphilic copolymerization with glycol CHS (gCHS) via strong ionic interactions. The copolymer loaded with MTX (MTX-gCHS-SA NPs) was found to have superior mechanical strength with enhanced sustained release pattern and reduced toxicity [86].

Some chemicals can function as both structuring and therapeutic agents to form CHS conjugations, with prolonged drug release and enhanced pharmacological effects. Chondroitin sulfate is an anionic glycosaminoglycan containing sulfate and carboxyl groups, which can form polyelectrolyte complex hydrogel membranes with CHS, mimicking an extracellular matrix structure and providing sustained drug release [87]. In addition, CHS-chondroitin sulfate NPs can reduce dosage and associated side effects of loaded drugs, due to the synergistic anti-arthritic effects of the glycosaminoglycan, which can regulate cartilage function by stimulating synthesis of proteoglycan and type II collagen [88]. Clodronate is a first-generation bisphosphonate with anti-inflammatory properties. Due to its structural analogy with tripolyphosphate, it can cause gelation of poloxamers and induce formation of sol-gel systems [89]. A matrix of poloxamer thermoreversible gels containing clodronate in CHS NPs was demonstrated with increased drug retention in joints, improved therapeutic indexes, and decreased side effects [90].

**Figure 5:**
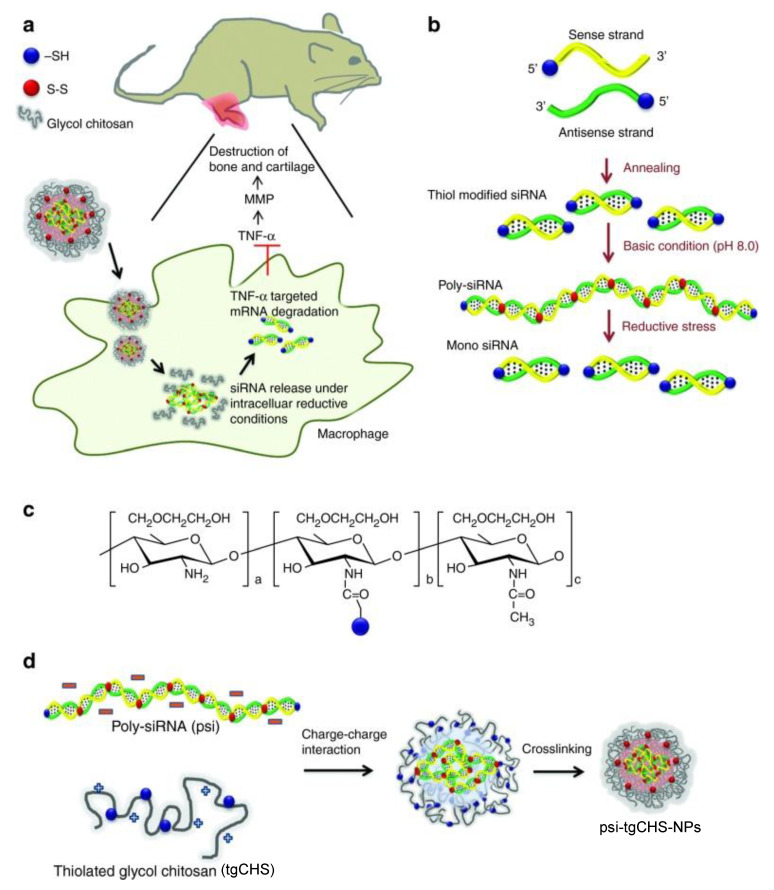
Schematic preparation and administration of an siRNA-loaded CHS nanosystem. (**a**) Uptake of psi-tgCHS-NPs into macrophage cells leading to tumor necrosis factor (TNF)-α gene knockdown. (**b**) Formation of poly-siRNA. (**c**) Synthesis of tgCHS polymers. (**d**) Complexation of poly-siRNA with tgCHS polymers [93]. Reproduced with permission from Lee et al., *Mol. Ther.*; published by Cell Press, 2014.

CHS nanosystems can achieve targeted drug delivery by enveloping specific nucleic acids. PSA-tmCHS NPs coated with decoy oligodeoxynucleotides (ODNs) specific to transcription factor NF-κB were found to increase cellular uptake. The NF-κB decoy ODNs mimicked the native DNA binding site of the transcription factor; by binding to NF-κB in the cytoplasm, the nucleic acid drug could prevent nuclear translocation and mitigate transcription of pro-inflammatory proteins [91]. The NP system could either act alone or be loaded with other therapeutics (e.g., MTX) to achieve anti-arthritic efficacy via a synergistic approach [92]. A nanocomplex of polymerized siRNA targeting TNF-α, a cytokine inducing chronic inflammation, conjugated with thiolated glycol CHA polymers (psi(TNF-α)-tgCHS NPs) has been designed to treat RA. The poly-siRNA conjugated with tgCHS via charge interactions and disulfide crosslinking reaction. The psi(TNF-α)-tgCHS NPs had high accumulation at the arthritic joint sites in CIA mice, and exhibited rapid cellular penetration and superior TNF-α gene silencing efficacy in macrophages (Figure 5) [93]. Similarly, a Notch1-targeted siRNA delivery system (psi(Notch1)-tgCHSNPs) successfully retarded RA statuses in vivo, by suppressing the synoviocyte-regulating Notch 1 signaling pathway without severe toxicity [94].

### 3.6. Folate and Folic Acid

Folate is a water-soluble B vitamin naturally occurring in various foods including green leafy vegetables, citrus fruits, and nuts, with functions of synthesizing nucleic acids and metabolizing amino acids. Folic acid (FA) is a synthetic form of folate that is used in fortified foods and most dietary supplements [95]. In RA, activated macrophages have been abundantly found in diseased synovia, overexpressing folate receptor β (FRβ) [96]. An opportunity is therefore provided for targeted delivery of both therapeutics and imaging agents by folate or FA-modified nanostructures.

NPs composed of a carrier structure, a drug substance, and folate/FA as surface modification can readily target activated macrophages in treating RA (Table 4). Fifth-generation poly(amidoamine) dendrimers (G5) can be employed as macromolecular drug deliverers because of their uniformity, biocompatibility, and capability to chemically couple multiple molecular entities to primary surface amino groups [97]. It was demonstrated that G5 NPs conjugated with FA and MTX beneficially suppressed inflammatory changes in arthritic animals [98]. Albumins normally bind and transport nutrients to cells throughout the body, with great potential to act as biodegradable and safe transporters for pharmaceuticals. Etoricoxib-loaded FA-conjugated bovine serum albumin NPs were shown to have high therapeutic effectiveness at a low dose [99]. Hybrid NP systems, such as NP-loaded liposomes (Figure 4H) and lipid–polymer hybrid nanoparticles (LPNPs; Figure 4I) are novel and robust drug delivery platforms. Application examples of the former system include folate-conjugated liposomes with co-entrapment of MTX and NF-kB siRNA-loaded CaP NPs [100]. The latter system comprises (1) a hydrophobic polymeric core encapsulating poorly water-soluble drugs, (2) an anti-biofouling hydrophilic polymeric shell, and (3) a lipid monomer at the core-shell interface retaining the drug in the core. There has been evidence of LPNPs loaded with small compounds or macromolecules in successful activated macrophage-targeted therapy [101].

**Figure 6:**
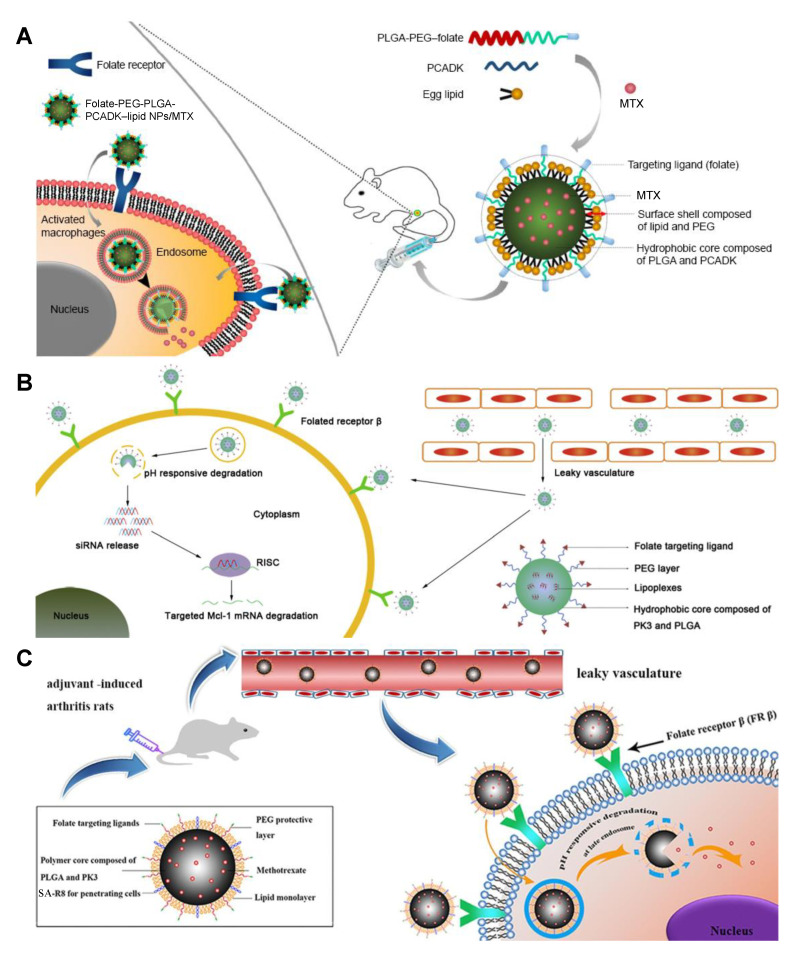
Schematic cellular uptake of representative folate/FA (folic acid)-modified nanoscopic drug delivery systems. (**A**) Folate-PEG-PLGA-PCADK–lipid NPs [103], reproduced with permission from Zhao et al., *Int. J. Nanomedicine*; published by Dove Medical Press, 2017; (**B**) Folate-PEG-PLGA-PK3 polymeric NPs [104], reproduced with permission from Sun et al., *Nanomedicine Nanotechnology, Biol. Med.*; published by Elsevier, 2019; (**C**) SA-R8-Folate-PEG-PLGA-PK3 lipid polymeric hybrid NPs [105], reproduced with permission from Zhao et al., *Eur. J. Pharm. Biopharm.*; published by Elsevier, 2018. FA, folic acid; PEG, polyethylene glycol; PLGA, poly(lactic-co-glycolic acid); PCADK, poly(cyclohexane-1,4-diylacetone dimethylene ketal); MTX, methotrexate; PK3, polyketal 3; SA, stearic acid.

Intelligent folate/FA-decorated nanosystems have been exploited in RA treatment, with additional materials responding to specific stimuli, e.g., pH level and redox potential. Since the pH in inflamed tissues (pH ~6.5), endosomes (pH ~5–6), and lysosomes (pH ~4–5) is significantly lower than that in blood circulation (pH ~7.4) [102], pH-responsive agents that degrade at acidic environments, such as polyketals (PKs) of poly(cyclohexane-1,4-diylacetone dimethylene ketal) (PCADK) and PK3, have been used as the pH-sensitive switch in RA drug delivery. An MTX-loaded pH-responsive LPNP system was designed using polyethylene glycol (PEG)–PLGA as a hydrophilic shell, FA conjugated on the shell as a targeting ligand, egg PC as an interface lipid, and PCADK and PLGA forming a hydrophobic core (Figure 6A) [103]. An Mcl-1 siRNA-encapsulated LPNP has similar construction except that PLGA and PK3 jointly formed the hydrophobic core, which confined lipoplexes being made of siRNA and 1,2-dioleyl-3-trimethylammonium propane (DOTAP), a cationic lipid (Figure 6B) [104]. The cell penetration effect of the target-specific LPNPs can be further enhanced by conjugating stearic acid-octa-arginine (SA-R8) on the particle surface (Figure 6C) [105]. Reactive oxygen species (ROS) have been found with significantly increased generation in the arthritic synovium under inflammatory stimulations [106], so ROS-responsive molecules can be used as triggers for anti-RA drug release as well as antioxidants balancing the redox state. ROS-responsive agents include synthetic molecules, e.g., 4-phenylboronic acid pinacol ester-conjugated cyclodextrin biomaterials (Oxi-αCD), which were reported forming DEX-loaded FA-Oxi-αCD NPs [107], and natural antioxidants, e.g., catalase, which was reported co-encapsulated with MTX in folate-anchored liposomes [108].

Many FR-targeted liposomes or nanomicelles have been designed as FA/folate covalently attached to a phospholipid anchor via a PEG linker, in order to achieve prolonged circulation time in vivo. For example, FA-PEG was conjugated on the surface of a double liposome, consisting of inner liposomes loaded with PRD and MTX, and an outer lipid bilayer which can impart safety to the inner liposomes against enzymatic degradation (Figure 4J) [109]. PSA has similar properties to PEG and is even more biodegradable; PSA therefore has great potential as a next generation of stealth biomacromolecule for nanomedicines. It was found that DEX loaded in FA-covered cholesterol—PSA nanomicelles demonstrated a 4–5-fold longer elimination half-life and a 2–3-fold higher bioavailability than commercial DEX injection [110]. Natural peptides can also be used as linkers between surface conjugation agents and phospholipids. Since mammalian pulmonary surfactant proteins can naturally promote self-assembly of phospholipids toward zero potential interface [111], a peptide derived from α-helical neck region of pulmonary surfactant-associated protein D (SP-D) was utilized to link FA on liposomes; the drug-loaded FA-SP-D-liposome was proven to be 2-fold more efficient in targeting FR-overexpressing Caco-2 cells than the free drugs (Figure 4F) [112].

Apart from targeted treatment of RA, FA-decorated NPs offer unique properties for magnetic resonance imaging (MRI) of the disease. Superparamagnetic iron oxide NPs modified with FA and diblock copolymers of PEG-polyacrylic acid (FA-PEG-PAA@SPIONPs) exhibited enhanced performance in RA diagnosis in vivo. The reasons include (1) SPIONPs with hydrodynamic diameters within 9 nm have high blood vessel penetration efficiency; (2) the PEG coating increased blood circulation stability of the iron oxide contrast agents; (3) PAA with proper length controlled the core size of the NPs; and (4) FA guided the NPs binding to activated macrophages in the synovium [113]. In another SPIONP design, the diblock copolymer was replaced by a conjugation of dextran and glucose, where dextran functioned as the polymeric material to stabilize the NPs, and glucose precipitated iron salts and subsequently reduced NP sizes. The detection signal was proven significantly enhanced by the coatings [114].

### 3.7. Black Pepper

AgNPs are known for their potential medical benefits, including anti-inflammatory activities [115]. Conventional physicochemical approaches for synthesizing AgNPs involve toxic stabilizers to prevent NP agglomeration, which can cause potential environmental and biological hazards [116]. Facile and eco-friendly synthesis of silver NPs can be assisted by food extracts, including aqueous extract of black pepper (*Piper nigrum*) seeds, which contain non-toxic and environmentally benign chemicals. It was postulated that the phytomolecules act as scaffolds/templates and bioreductants to react with metal ions in the following three steps: (1) piperine or proteins from the black pepper extract can trap the silver ions via electrostatic interactions; (2) the pepper proteins can reduce the silver ions, subsequently change in secondary structures and form silver nuclei; (3) the silver nuclei can grow along with further silver ion reduction and accumulation on the nuclei [117].

The anti-arthritic effects of the phyto-stabilized AgNPs using black pepper seed extract was depicted by reduction of paw edema and alleviation of histopathological changes in inflamed joints of AIA rats (Table 4). Such therapeutic activity was discovered to be mediated by inhibition of TNF-α and NF-kβ. Moreover, it was found that the black pepper extract-stabilized AgNPs prepared via the green method showed more evident anti-arthritic potency than commercial AgNPs synthesized with conventional hazardous methods, indicating that the stabilizing phytochemicals from black pepper, especially the predominated alkaloid piperine, also exert therapeutic contributions against RA [118].

## 4. Conclusions and Perspectives

Many food-derived small chemicals, especially polyphenols and flavonoids, have been reported with anti-arthritic bioactivities but limited aqueous solubility and stability. To improve their physicochemical properties as well as therapeutic efficacies, they have been incorporated into different nano-drug delivery systems for RA treatment. Such natural compounds have a large variety of chemical scaffolds and have long-term evidence of safety, showing advantages over most synthetic agents, and therefore can provide inspirations on novel anti-RA drug development. Moreover, in comparison to single drug administration, co-encapsulation of multiple food components, e.g., curcumin and resveratrol, sometimes exhibited elevated anti-arthritic effects. It is thus promising that more combinations of food-derived bioactive compounds can be delivered with enhanced therapeutic performance. Exploration on anti-RA efficacies of food components have given study orientations on their action mechanisms as well as toxicity and efficacy profiles, further contributing to developing the therapeutic chemicals.

Larger molecules from foods, including fatty substances, polysaccharides, and amine-containing compounds, have been widely used as building blocks of various nanostructures for drug delivery. Nanoformulations using food components as structuring agents have been discovered with enhanced drug adsorption and release as well as reduced side effects via different delivery routes. Therefore, such delivery systems show high promise to be employed with current RA medications to achieve elevated therapeutic performances. In addition, it is discovered that some structuring agents, e.g., emu oil and components from black pepper extract, have anti-inflammatory potencies and contribute to the overall therapeutic effects of the drug delivery system. Therefore, multi-functional food ingredients show great potential in nano-drug delivery systems, where therapeutic molecules can self-assemble into nanostructures ready for direct administration.

Food-derived nanoscopic drug delivery systems provide safe, effective treatment strategies for RA, a serious and chronic autoimmune inflammatory disease. Both small and large molecules derived from foods have been demonstrated to play significant roles in nanosystems. However, much current research is still limited to preclinical studies. As evidence accumulates, clinical studies will be the next step in bringing these RA therapeutic approaches with higher efficacy and fewer side effects into medical practice.

## Figures and Tables

**Table 1:** Representative applications of food components as delivered anti-RA drugs.

Food ingredient	Nano system	Model	Dose	Pharmacological Effects	Ref.
Curcumin	NEs	AIA rats	50 mg/kg/d; *p.o.*; 14 days	Paw swelling↓↓ ^a^; TNF-α and IL-1β decreased	[13]
Curcumin	Solid lipid nanoparticles	AIA rats	10 and 30 mg/kg/d; *p.o.*; 14 days	Joint hyperalgesia↓↓↓ ^a^; mobility score↑↑↑↑ ^a^; joint stiffness↓↓↓↓ ^a^; paw volume↓↓↓↓ ^a^; radiological score decreased	[20]
Curcumin	Nanomicelles	RA patients	40 mg/*tid.*; *p.o.*; 12 weeks	Disease activity score of joints↓↓↓↓ ^b^; tender joint count↓↓↓ ^b^; swollen joint count↓↓ ^b^	[24]
Curcumin	NEG	AIA rats	25.71 mg/kg/*bid.*; *top.*; 28 days	Body weight ↑↑ ^a^; tibiotarsal joint thickness↓ ^a^; IL-1β, TNF-α↓↓ ^a^; paw volume decreased; histopathological changes alleviated	[26]
Curcumin	NEG	Carrageenan-induced paw edema; rat skin	30 mg/kg; *top.*	Anti-inflammatory effects↑↑↑ ^d^; skin permeation increased	[27]
Curcumin	NEG	Carrageenan-induced paw edema; AIA rats		Paw edema↓↓↓ ^a^; body weight↑↑↑ ^a^; paw volume↓↓↓ ^a^; motor incoordination↓↓↓ ^a^; arthritic clinical score↓↓↓ ^a^; synovium TNF-α, IL-6, IL-1β ↓↓↓ ^a^; serum TNF-α, IL-6 ↓↓↓ ^a^; radiological score↓↓↓ ^a^; histopathological changes alleviated	[28]
Curcumin and Resveratrol	Lipid core nanocapsules	AIA rats	1.75 mg/kg/*bid*; *i.p.*; 8 days	Paw edema↓ ^d^; synovial fibrosis ↓ ^a^; cartilage loss↓ ^a^; bone loss↓ ^a^; histopathological changes alleviated	[31]
Curcumin	Nanomicelles	CIA rats	33.6 μg; IA inj.	Paw edema↓ ^b^; TNF-α, IL-1, VEGF↓ ^a^; histopathological changes alleviated	[32]
Resveratrol	Nanomicelles	AIA rats	1 mg/mL/week; IA inj.; 14 days	Knee swelling↓ ^a^; TNF-α↓ ^a^; histopathological changes alleviated	[37]
sveratrol	QRu-PLGA-DS NPs	RAW 264.7 cells; CIA mice		TNF-α, IL-1β, IL-6↓↓ ^a^; IL-4, IL-10, TGF-β↑↑ ^a^; histopathological changes alleviated	[38]
Quercetin	TGA-CdTe QDs	AIA rats	0.2 and 0.4 mg/kg/d; *p.o.*; 21 days	Inflammation reduced; cartilage regeneration improved; histopathological changes alleviated	[43]
Quercetin	NEG	AIA rats	10 mg/*bid*; *top.*; 28 days	TNF-α↓ ^c^; arthritic index↓ ^a^; stiffness score↓ ^a^; paw circumference↓ ^a^; rheumatoid factor↓ ^a^	[44]
Quercetin	PCL microspheres	HIG-82 cells, rats	0.1 mL; IA inj.	Synovial macrophage proliferation reduced; controlled release of quercetin in the joint cavity for more than 30 days	[45]
Hesperidin	AgNPs	AIA rats	1mg/kg; *p.o.*; 14 days	Arthritic score↓↓↓ ^a^; paw swelling↓↓↓ ^a^; TLR-2, TLR-4↓↓↓ ^a^	[48]

^a^ Compared with model group; ^b^ compared with before treatment; ^c^ compared with free drug administration; ^d^ compared with vehicle control. Single arrow, *p* < 0.05; double arrows, *p* < 0.01; triple arrows, *p* < 0.001; four arrows, *p* < 0.0001. Abbreviations: NEs, nanoemulsions; AIA, adjuvant-induced arthritis; RA, rheumatoid arthritis; NEG, nanoemulsion gel; CIA, collagen-induced arthritis; VEGF, vascular endothelial growth factor; IA, intra-articular; QRu-DS NPs, quadrilateral ruthenium (core)-dextran sulfate-modified poly (lactic-co-glycolic acid) (shell) nanoparticles; TGA-CdTe QDs, thio glycolic acid-capped cadmium telluride quantum dots; PCL, polycaprolactone; AgNPs, silver nanoparticles.

**Table 2:** Representative fabrications of anti-RA nanocarriers using dietary oils and lecithin.

Food Material	Nano System	Loaded Drug	Model	Dose	Pharmacological Effects	Ref.
Soybean oil	NEs	Curcumin	AIA rats	50 mg/kg/d; *p.o.*; 14 days	Paw swelling↓↓ ^a^; TNF-α and IL-1β decreased	[13]
Soybean oil	NEs	Oily mixture of camphor, menthol and methyl salicylate	Rat skin	5% camphor, 5% menthol, and 5% methyl salicylate	Permeation rates increased	[54]
Grape seed oil	Lipid core nanocapsules	Curcumin, resveratrol	AIA rats	1.75 mg/kg/*bid*; *i.p.*; 8 days	Paw edema↓ ^d^; synovial fibrosis↓ ^a^; cartilage loss↓ ^a^; bone loss↓ ^a^; radiological score decreased	[31]
Emu oil	NEG	Curcumin	Carrageenan induced paw edema, AIA rats	*Top.*	Paw edema↓↓↓ ^a^; body weight↑↑↑ ^a^; motor incoordination↓↓↓ ^a^; arthritic clinical score↓↓↓ ^a^; synovial TNF-α, IL-6, IL-1β↓↓↓ ^a^; serum TNF-α, IL-6 ↓↓↓ ^a^; radiological score↓↓↓ ^a^; histopathological changes alleviated	[28]
Soy lecithin	Liposomes	Triptolide	CIA mice	200 mg/kg/d; *top.*; 25 days	Pannus number↓↓ ^a^; histopathological changes alleviated	[64]
Soy lecithin	NSSLs	MPS; BMS	AIA rats	①At early stage: 10 mg/kg; *i.v.*; 2 times (NSSLs-MPS) or 3 times (NSSLs-BMS). ②At late stage: 10 mg/kg (NSSLs-MPS) or 5 mg/kg (NSSLs-BMS), *i.v.*, 2 times	①At early stage: arthritis score↓↓↓ ^a^ ②At late stage: arthritis score↓ ^a^	[66]
Soy lecithin	NSSLs	MPS; BMS	AIA rats	NSSLs-MPS: ①10 mg/kg/week; *i.v.*; 3 times; ②10 mg/kg/week; *s.c.*; 2 times; ③2 mg/kg/week; *s.c.*; 3 times; ④1 or 10 mg/kg; *i.v.*; 1 time; ⑤10 mg/kg; *i.v.* NSSLs-BMS: 1 mg/kg/week; *s.c.*; 3 times	NSSLs-MPS: ①arthritis score↓ ^a^; ②arthritis score↓↓ ^a^; ③arthritis score↓↓ ^a^;④arthritis score↓ ^a^; ⑤splenocyte IL-6↓↓ ^a^, IL-10↓ ^a^, INF-γ↓ ^a^; serum IL-6↓ ^a^ NSSLs-BMS: arthritis score↓↓ ^a^	[67]
Soy lecithin	Sialic acid-modified liposomes	Dexamethasane palmitate	AIA rats	0.9 mg/kg/3 days; *i.v.*; 5 times	Paw thickness↓↓ ^a^; joint score↓↓↓ ^a^; IL-1β↓ ^a^, TNF-α↓↓↓ ^a^; histological scores↓↓ ^a^	[70]
Egg lecithin	Liposomes	Indomethacin	AIA rats	3 mg/kg/d; *i.p.*; 15 days	Edema volume↓↓ ^c^; Ulcerogenicity↓↓ ^c^	[71]
Egg lecithin	Liposome hydrogel patch	Triptolide	CIA rats	20 mg/kg; *top.*; 4 weeks; 40 mg/kg; *top.*; 4 weeks	20 mg/kg: joint swelling↓↓ ^a^; IL-1β↓ ^a^, IL-6↓↓↓ ^a^; Flk-1↓↓↓ ^a^; Flt-4↓↓↓ ^a^; HIF-1α↓ ^a^ 40 mg/kg: joint swelling↓↓↓ ^a^; IL-1β, IL-6↓↓↓ ^a^; Flk-1↓↓↓ ^a^; Flt-4↓↓↓ ^a^; HIF-1↓↓ ^a^	[72]
Egg lecithin	Ultradeformable liposomal gel	MTX	AIA rats	0.5 mg/kg/3 days; *top.*; 8 times	Paw edema volume↓↓↓ ^a^; body weight↑↑↑ ^a^; paw histological score↓↓ ^a^; leukocyte infiltration↓ ^a^; neutrophils in paw tissues↓ ^a^; TNF-a↓ ^a^; IL-1β↓ ^a^	[74]
Egg lecithin	WSs	TNF-α siRNA	CIA mice	10 μg/body; three times a week; *i.v.*;11 days	Arthritis incidance↓; arthritis score↓; paw thickness↓; TNF-α↓	[76]
Egg lecithin	Large unilamellar vesicles	APO2L/TRAIL	Antigen-induced arthritic rabbits	5 μg; 10 μg	5 μg: Knee lateral diameter ↓ ^a^; inflammation↓ ^a^; synovial hyperplasia↓ ^a^ 10 μg: Knee lateral diameter↓↓ ^a^	[78]

^a^ Compared with model group; ^c^ compared with free drug administration; ^d^ compared with vehicle control. Single arrow, *p* < 0.05; double arrows, *p* < 0.01; triple arrows, *p* < 0.001. Abbreviations: NEs, nanoemulsions; AIA, adjuvant-induced arthritis; NEG, nanoemulsion gel; CIA, collagen-induced arthritis; NSSLs, sterically stabilized nanoliposomes; MPS, methylprednisolone hemisuccinate; BMS, betamethasone hemisuccinate; MTX, methotrexate; WS, wrapsome.

**Table 3:** Representative fabrications of anti-RA nanocarriers using chitosan.

Food material	Nano system	Loaded drug	Model	Dose	Pharmacological effects	Ref.
CHS	CHS NPs	Betamethasone sodium phosphate	Rats	1 and 2 mg/kg	Without any toxic effect on vital organs	[82]
CHS	CHS NPs	Embelin	AIA rats	25 and 50 mg/kg/d; *p.o.*; 14days	25 and 50 mg/kg: arthritic score and paw swelling decreased 25 mg/kg: TNF-α↓↓ ^a^; IL-1β↓ ^a^; IL-6↓ ^a^50 mg/kg: TNF-α; IL-1β; IL-6↓↓↓ ^a^	[83]
CHS	CHS NPs	Zinc gluconate	CIA rats	112.93 mg/kg; *i.p.*	Serum TNF-α↓↓ ^a^, IL-1β↓↓↓ ^a^; joint synovial IL-6↓↓↓ ^a^, TNF-α↓↓↓ ^a^, iNOS↓↓↓ ^a^; histopathological changes alleviated	[84]
CHS	Polysialic acid-trimethyl CHS NPs	DEX; MTX	SW-982 cells	1.0 mg/mL	DEX: IL-6↓ ^a,d^; IL-8↓ ^a^ MTX: IL-8↓ ^a^	[85]
CHS	Glycol CHS-steric acid NPs	MTX	AIA mice	0.5 mg/kg/3d; *i.v.*	Arthritis score↓↓ ^a^; paw thickness↓↓ ^a^; IL-1β, TNF-a↓ ^c^	[86]
CHS	CHS-chondroitin sulfate NP-loaded argan oil emulsion gel	Ketoprofen	Mice skin		Compared with marketed gel, skin permeability↑↑↑↑; compared with NP-loaded gel, skin permeability↑↑	[87]
CHS	CHS-NP-loaded poloxamer gel	Clodronate	THP1 cells	1, 2, and 4 μg/mL	IL-8 and IL-1β decreased	[90]
CHS	Thiolated glycol CHS NPs	Polymerized siRNA	RAW 264.7 cells; CIA mice	50 μg; *i.v.*	TNF-α in RAW 264.7 cells decreased; TNF-α in serum and arthritic joints↓ ^b^; arthritic score, paw thickness decreased; bone erosions in paws and ankle joints decreased	[93]
CHS	Thiolated glycol CHS NPs	Polymerized siRNA	RAW 264.7 cells; CIA mice		Notch1 in RAW 264.7 cells ↓↓↓ ^a^; synovial inflammation↓↓↓ ^a^; cartilage erosion↓↓ ^a^; neutrophil infiltration↓↓↓ ^a^; clinical score, bone damage decreased	[94]

^a^ Compared with model group; ^b^ compared with before; ^d^ compared with vehicle control. Single arrow, *p* < 0.05; double arrows, *p* < 0.01; triple arrows, *p* < 0.001; four arrows, *p* < 0.0001. Abbreviations: CHS, Chitosan; DEX, dexamethasone; MTX, methotrexate; NPs, nanoparticles; AIA, adjuvant-induced arthritic; CIA, collagen-induced arthritic; siRNA, small interfering RNA.

**Table 4:** Representative fabrications of anti-RA nanocarriers using folate, folic acid and black pepper extract.

Food material	Nano system	Loaded drug	Model	Dose	Pharmacological effects	Ref.
Folate	Folate-conjugated albumin nanoparticles	Etoricoxib	Carrageenan induced arthritis	5 mg/kg; *i.v.*	Inhibition of edema increased	[99]
Folate	Folate-conjugated liposomes	NF-kB-targeted siRNA; MTX	RAW 264.7 cells; arthritic mice	*i.v.*	Cellular uptake increased; paw thickness, arthritic scores, TNF-α and IL-1β decreased	[100]
Folate	Folate-liposomes	MTX; catalase	RAW 264.7 cells; CIA mice	1 mg/kg/2d; *i.v.*; 5 times	Cellular uptake increased; arthritis score↓↓ ^a^; paw thickness↓↓ ^a^; body weight↑↑ ^a^; TNF-α↓↓↓ ^a^, IL-1β↓↓↓ ^a^	[108]
Folate	Folate conjugated double liposomes	PRD; MTX	CIA rats	1 mg/kg; *i.v.*	Inhibition of edema increased	[109]
Folate	FA-PEG-PLGA-PCADK-lipid NPs	MTX	RAW 264.7 cells; AIA rats	257 μg/kg/2d; *i.v.*	Cellular uptake increased; clinical score↓↓ ^c^; paw thickness↓↓ ^c^; TNF-α↓↓↓ ^c^, IL-6↓↓ ^c^; histopathological changes alleviated	[103]
Folate	FA-PEG-PLGA-PK3 NPs	Mcl-1 siRNA	RAW 264.7 cells; AIA rats	4 nmol/kg/2d; *i.v.*; three times	Cellular uptake increased; clinical score↓↓↓ ^a^; paw thickness↓↓↓ ^a^; TNF-α↓↓↓ ^a^, IL-6↓↓^a^, IL-1β↓↓ ^a^; histopathological changes alleviated	[104]
Folate	SA-R8-FA-PEG-PLGA-lipid polymeric hybrid NPs	MTX	RAW 264.7 cells; AIA rats		Cellular uptake increased; clinical score↓↓↓ ^c^; paw thickness↓↓↓ ^c^; TNF-α↓↓ ^c^, IL-6↓↓↓^c^, IL-1β↓↓↓ ^c^; histopathological changes alleviated	[105]
FA	FA-Oxi-αCD NPs	DEX	RAW 264.7 cells; CIA mice	1.25mg/kg/4d; *i.v.*	TNF-a↓ ^a^; arthritis index↓ ^a^; paw thickness↓ ^a^; synovial inflammation↓ ^a^; cartilage erosion ^a^; histopathological changes alleviated	[107]
FA	FA-polysialic acid- cholesteryl chloroformate micelles	DEX	RAW 264.7 cells; AIA mice	RAW 264.7 cells: 0.1mg/mL AIA mice: 0.02mg/ 2d; 10 days	RAW 264.7 cells: TNF-a, IL-6 decreased AIA mice: Paw thickness, clinical index, TNF-a, IL-6 decreased	[110]
FA	FA-SP-D-liposomes	Celecoxib	Caco-2 cells		Compared with celecoxib-loaded liposomes, COX-2↓	[112]
FA	FA-PEG-PAA@SPIONPs		RAW 264.7 cells; Antigen induced arthritic rats	5 mg/kg; *i.v.*	Cellular uptake increased; MRI enhanced in diagnosis	[113]
FA	FA-glucose-dextran-SPIONPs		RAW 264.7 cells; Antigen induced arthritic rats	25 mg/kg; *i.v.*	Cellular uptake increased; MRI enhanced in diagnosis and therapy response	[114]
Black pepper extract	Phyto-stabilised AgNPs		AIA rats	25 and 50 mg/kg/2d; *i.p.*; 6 times	Paw volume↓ ^a^; gait score↓ ^a^; histopathologic score↓ ^a^; histopathological changes alleviated	[118]

^a^ Compared with model group; ^b^ compared with before treatment; ^c^ compared with free drug administration. Single arrow, *p* < 0.05; double arrows, *p* < 0.01; triple arrows, *p* < 0.001. Abbreviations: MTX, methotrexate; PRD, prednisolone; CIA, collagen-induced arthritic; FA, folic acid; G5, fifth-generation poly(amidoamine) dendrimers; NPs, nanoparticles; PEG, polyethylene glycol; PLGA, poly (lactic-co-glycolic acid); PCADK, poly(cyclohexane-1,4-diylacetone dimethylene ketal); PK3, polyketals 3; Mcl-1, Myeloid cell leukemia-1; AIA, adjuvant-induced arthritis; SA, stearic acid; Oxi-αcd, 4-phenylboronic acid pinacol ester-conjugated cyclodextrin biomaterials; DEX, dexamethasone; SP-D, surfactant-associated protein D; SPIONPs, superparamagnetic iron oxide nanoparticles; MRI, magnetic resonance imaging; AgNPs, silver nanoparticles.
